# Effects of a virtual supportive program on the knowledge of mothers of preterm infants and their bonding

**DOI:** 10.1002/imhj.70013

**Published:** 2025-04-24

**Authors:** Supaporn Pusri, Sudaporn Payakkaraung, Wanlaya Thampanichawat

**Affiliations:** ^1^ Master Program in Nursing Science (International Program), Faculty of Nursing Mahidol University Bangkok Thailand; ^2^ Faculty of Nursing Mahidol University Bangkok Thailand

**Keywords:** knowledge, mother–infant bonding, preterm cues, preterm infant, virtual support, المعرفة، الترابط بين الأم والرضيع، إشارات الخدج، الرضع الخدج، الدعم الافتراضي, 认知, 母婴关系, 早产儿行为线索, 早产儿, 虚拟支持, Connaissances, lien entre la mère et le nourrisson, indices des nourrissons prématurés, nourrisson prématuré, soutien virtuel, Wissen, Mutter‐Kind‐Bindung, Verhaltensweisen Frühgeborener, Frühgeburten, virtuelle Unterstützung, 知識、母‐子ボンディング、早産児の手がかり、早産児、バーチャル支援, conocimiento, unión afectiva madre‐infante, señales prematuras, infantes prematuros, apoyo virtual

## Abstract

This quasi‐experimental study examined the effect of a virtual supportive program on knowledge among mothers of preterm infants and their bond. Mothers of preterm infants are at risk of becoming emotionally detached from their infants because of immediate separation and the lack of support during the early postpartum period, especially in mothers who cannot visit their infant regularly. To the best of our knowledge, no study in Thailand has examined the effects of virtual support for these mothers, as most scholars have focused on promoting mother–infant interaction to improve bonding and enhancing mothers’ knowledge about their infant before discharge. This study recruited 74 mothers with preterm infants receiving neonatal intensive and intermediate care in Thailand. The participants were matched to mothers according to their infants’ gestational ages and assigned into the following two groups: experimental group receiving virtual support, and control group receiving routine care. The results showed that the virtual supportive program significantly improved mothers’ knowledge on preterm infants’ cues and maternal roles as well as mother–infant bonding. Consistent support during the first week of visitation was essential, especially in providing information about premature infants and emotional support, to improve mother–infant bonding.

## ملخص

تناولت هذه الدراسة شبه التجريبية تأثير برنامج افتراضي داعم على معرفة أمهات الأطفال الخدج وارتباطهن. إن أمهات الأطفال الخدج معرضات لخطر الانفصال العاطفي عن أطفالهن الرضع بسبب الانفصال الفوري ونقص الدعم خلال فترة ما بعد الولادة المبكرة، خاصة لدى الأمهات اللاتي لا يستطعن زيارة أطفالهن الرضع بانتظام. على حد علمنا، لم تفحص أي دراسة في تايلاند آثار الدعم الافتراضي لهؤلاء الأمهات، حيث ركز معظم العلماء على تعزيز التفاعل بين الأم والرضيع لتحسين الترابط وتعزيز معرفة الأمهات برضيعهن قبل الخروج من المستشفى. سجلت هذه الدراسة 74 من الأمهات اللاتي لديهن أطفال خدج يتلقون رعاية مكثفة ومتوسطة لحديثي الولادة في تايلاند. تمت مطابقة المشاركات مع الأمهات وفقاً لأعمار حمل أطفالهن الرضع وتم توزيعهن على المجموعتين التاليتين: المجموعة التجريبية التي تتلقى الدعم الافتراضي، والمجموعة الضابطة التي تتلقى الرعاية الروتينية. أظهرت النتائج أن البرنامج الداعم الافتراضي حسّن بشكل كبير معرفة الأمهات بإشارات الأطفال الخدج وأدوار الأمهات بالإضافة إلى الترابط بين الأم والرضيع. كان الدعم المستمر خلال الأسبوع الأول من الزيارة ضروريًا، خاصة في توفير المعلومات عن الأطفال الخدج والدعم العاطفي لتحسين الترابط بين الأم والرضيع.

## 抽象的

摘 要:这项准实验研究探讨了一项虚拟支持计划对早产儿母亲知识水平及其母婴关系的影响。由于产后立即与婴儿分离以及缺乏支持, 早产儿母亲, 尤其是无法定期探视婴儿的母亲, 面临与婴儿情感疏离的风险。据我们所知, 泰国尚未有研究探讨虚拟支持对这些母亲的影响, 因为大多数学者关注的是通过促进母婴互动来改善情感联结, 并在出院前提升母亲对婴儿的认知。本研究招募了74名在泰国接受新生儿重症监护和中级护理的早产儿母亲。研究根据婴儿的胎龄匹配母亲, 并分为以下两组:实验组接受虚拟支持;对照组接受常规护理。结果显示, 虚拟支持计划显著提高了母亲对早产儿行为线索和母亲角色的认知, 并改善了母婴关系。研究强调, 在母亲首次探视的第一周内提供持续支持, 尤其是关于早产儿信息的教育和情感支持, 对于促进母婴关系至关重要。

## abstraite

Cette étude quasi expérimentale a examiné l'effet d'un programme de soutien virtuel sur les connaissances des mères de bébés prématurés et leur lien. Les mères de bébés prématurés sont à risque de détachement émotionnel envers leurs bébés en raison de la séparation immédiate et du manque de soutien au début de la période postpartum, surtout chez les mères qui ne peuvent pas rendre visite à leur bébé régulièrement. À notre connaissance, aucune étude en Thaïlande n'a examiné les effets du soutien virtuel pour ces mères, car la plupart des chercheurs se sont jusqu'ici concentrés sur la promotion de l'interaction mère‐enfant dans le but d'améliorer le lien et de renforcer les connaissances des mères au sujet de leur bébé avant qu'il ne quitte l'hôpital. Cette étude a recruté 74 mères avec des bébés prématurés recevant des soins néonatals intensifs et des soins intermédiaires en Thaïlande. Les participantes étaient ces mères et des mères contrôle présentant les mêmes caractéristiques d’âge gestationnel de leur enfant. Elles ont été réparties en deux groupes: le groupe expérimental recevant un soutien virtuel; et le groupe témoin recevant des soins de routine. Les résultats ont montré que le programme de soutien virtuel améliorait considérablement les connaissances des mères sur les indices présentés par les bébés prématurés et sur les rôles maternels, ainsi que sur la relation mère‐enfant. Un soutien constant durant la première semaine de visite s'est avéré essentiel, en particulier pour fournir des renseignements sur les prématurés et du soutien émotionnel afin d'améliorer le lien entre la mère et le bébé.

## Abstrakt

Diese quasi‐experimentelle Studie untersuchte die Auswirkungen eines virtuellen Unterstützungsprogramms auf das Wissen von Müttern von Frühgeborenen und deren Bindung. Mütter von Frühgeborenen sind dem Risiko ausgesetzt, sich aufgrund der sofortigen Trennung und des Mangels an Unterstützung in der frühen postpartalen Phase emotional von ihrem Kind zu entfremden, insbesondere wenn sie ihr Kind nicht regelmäßig besuchen können. Soweit wir wissen, gibt es in Thailand bisher keine Studie, die die Auswirkungen virtueller Unterstützung für diese Mütter untersucht hat, da sich die meisten Forschenden auf die Förderung der Mutter‐Kind‐Interaktion zur Stärkung der Bindung und auf die Verbesserung des Wissens der Mütter über ihr Kind vor der Entlassung konzentriert haben. Für diese Studie wurden 74 Mütter mit Frühgeborenen rekrutiert, die in Thailand eine neonatale Intensiv‐ oder Intensivüberwachungspflege erhalten. Die Mütter wurden entsprechend dem Gestationsalter ihrer Kinder gematcht und in die folgenden zwei Gruppen eingeteilt: die Versuchsgruppe, die virtuelle Unterstützung erhielt, und die Kontrollgruppe, die Routinepflege erhielt. Die Ergebnisse zeigten, dass das virtuelle Unterstützungsprogramm das Wissen der Mütter über die Verhaltensweisen ihrer Frühgeborenen und die mütterliche Rolle sowie die Mutter‐Kind‐Bindung deutlich verbesserte. Eine konsistente Unterstützung während der ersten Besuchswoche war für die Verbesserung der Mutter‐Kind‐Bindung von entscheidender Bedeutung, insbesondere durch die Bereitstellung von Informationen über Frühgeborene und emotionale Unterstützung.

## 抽象的な

この準実験的研究では、バーチャル支援プログラムが早産児の母親の知識とその母子のボンディングに及ぼす効果を検討した。早産児の母親は、産後すぐの分離や産褥期のサポート不足のため、特に定期的に乳児と面会できない母親においては、乳児から情緒的剥脱の危険性がある。私たちの知る限り、タイではこのような母親に対するバーチャル支援の効果を検討した研究はない。ほとんどの研究者は、ボンディングを深めるために母子の相互作用を促進したり、退院前に乳児に関する母親の知識を高めたりすることに焦点を当てている。本研究では、タイで新生児集中的及び中間的ケアを受けている早産児の母親74人を採用した。参加者は、乳児の在胎週数に応じて母親をマッチングし、以下の2群に分けた。すなわち、バーチャル支援を受けている実験群と日常的なケアを受けている対照群。その結果、バーチャル支援プログラムは、母‐子ボンディングだけでなく、早産児の手がかりについてや母親の役割に関する知識を有意に改善することが示された。母–子のボンディングを改善するためには、面会開始後最初の1週間の一貫した支援、特に未熟児に関する情報の提供と精神的支援が不可欠であった。

## Resumen

Este estudio cuasiexperimental examinó el efecto de un programa virtual de apoyo sobre el conocimiento entre madres de infantes nacidos prematuramente y su unión afectiva. Las madres de infantes prematuros se encuentran bajo riesgo de llegar a estar emocionalmente despegadas de sus infantes a causa de la inmediata separación y la falta de apoyo durante el temprano período del postparto, especialmente en el caso de madres que no pueden visitar a sus infantes con regularidad. Hasta donde tenemos conocimiento, no hay estudio en Tailandia que haya examinado los efectos del apoyo virtual para estas madres, ya que la mayoría de los investigadores se han enfocado en promover la interacción madre‐infante para mejorar la unión afectiva y aumentar el conocimiento de las madres acerca de sus infantes antes de que se les dé de alta. Este estudio reclutó 74 madres con infantes prematuros que recibían cuidado neonatal intensivo e intermedio en Tailandia. A las participantes se les aparejó con madres de acuerdo con las edades gestacionales de sus infantes y se les asignó en los siguientes dos grupos: grupo experimental que recibía apoyo virtual; grupo de control que recibía el cuidado de rutina. Los resultados muestran que el programa virtual de apoyo mejoró significativamente el conocimiento de las madres acerca de las señales de infantes prematuros y los papeles maternos, así como también la unión afectiva madre‐infante. El apoyo consistente durante la primera semana de visita fue esencial, especialmente en cuanto a aportar información acerca de infantes prematuros y apoyo emocional, para mejorar la unión afectiva madre‐infante.

## 1 INTRODUCTION

Premature infants often require extended hospital stays, resulting in separation from their parents and potentially impacting the developing parent–child relationship. In Thailand, an estimated 40,000 premature infants require hospitalization for specialized care each year, leading to nearly 100% bed occupancy (Department of Health, Ministry of Public Health, Thailand, 2023). Prolonged hospitalization negatively affects mothers’ ability to touch, feed, and comfort their infants; these activities crucial for fostering the maternal attachment bond (Feldman et al., 1999). Before the coronavirus disease 2019 (COVID‐19) pandemic, nurses in Thailand actively encouraged mothers to participate in the care of their premature infants while admitted in the neonatal intensive care unit (NICU). This included regular visits, diapering, bathing, providing breast milk, and engaging in kangaroo care, which enhanced mothers’ caretaking knowledge and strengthened the parent–infant relationship (Lebel et al., 2022). However, during the pandemic, it became considerably more challenging for mothers to spend time with their infants in the NICU. Mothers could only drop off breast milk and receive updates about their infants by telephone. In‐person visits required a negative COVID‐19 test result, which was cost‐prohibitive for many. Additionally, many mothers faced other barriers, such as needing to return to their villages to work or care for other children, making it even more difficult to visit the NICU. Nurses reported a decline in the number of mothers delivering breast milk and visiting their infants during this period. These restrictions caused heightened stress among mothers due to limited visitation opportunities, reduced chances to provide direct care, and inconsistent communication and support regarding their infants’ conditions (Vance et al., 2021; Yance et al., 2023). This challenge persists post‐pandemic. To support maternal knowledge and bonding with their infants in the NICU, we developed a remote intervention (virtual supportive program: bonding with preterm infants). The present study aimed to evaluate the intervention's impact on maternal knowledge of infant cues, their role during hospitalization, and the mother–infant bond.

### 1.1 Separation in the postpartum period and maternal caregiving knowledge

The understanding of infants’ cues by mothers is crucial for recognizing and addressing their needs, including hunger, tiredness, discomfort, and overstimulation. This understanding is essential for the infants’ survival and overall development. An appropriate response to these cues, along with the mother's knowledge of her role, is vital for providing consistent care, which lays the foundation for a strong bond between mothers and infants. Meeting the infants’ needs fosters trust, emotional security, self‐regulation, and cognitive growth, contributing to positive social behavior in the future (Als, 1982; Klaus & Kennell., 1976). Mothers need sufficient time and opportunities to be with their infants to learn and understand their cues, as well as the nuances of their maternal roles in caring for their premature infants. Increased time spent with their infants and involvement in infant care are associated with emotional closeness and the development of the mother–infant relationship (Lebel et al., 2022). The early postpartum period is a transitional time from pregnancy to motherhood, when mothers begin to learn about their new roles as a mother. However, mothers of preterm infants often experience heightened stress, including the inability to help and protect their child from painful procedure, as well as challenges with feeding and providing care. These stresses complicate their ability to understand and navigate their maternal roles and develop a strong relationship with their infants (Sikorova & Kucova, 2012).

**Key Findings**

1. Information on infant cues and emotional support is essential for improving bonding between mothers and their preterm infants in the hospital.
2. Early and consistent support is a crucial foundation for developing bonding between mothers and their preterm infants.
3. Mothers can improve their understanding of their preterm infants through the use of illustration and written information that they can review, in addition to bedside demonstrations.

**Statement of Relevance**

We investigated infant and early childhood mental health by exploring an alternative intervention to improve emotional closeness between mothers and their preterm infants. Our virtual supportive program provided mothers with consistent support, which is essential in building the foundation for mother–infant bonding, especially for those struggling to visit their infants regularly. It also facilitates mother–infant interaction by verifying mothers’ knowledge on preterm infants’ cues and their roles, constituting the foundation of appropriate mother–infant interaction in the future.

Separation from their infants limits mothers’ opportunities to provide care, leading to unclear roles since they do not have babies to care for. Additionally, the experiences with premature infants differ considerably from the mother's expectations during the pregnancy. Mothers often hold a more pessimistic view of their babies, which is particularly due their small size, medical condition, and associated equipment used, which can create a feeling of disconnection (Eamkusolkit et al., 2017; Fuertes et al., 2011). As a result, mothers perceive themselves as inadequate caregivers, feeling unable to meet their infants’ most basic needs and lacking autonomy in making decisions regarding their care. Understanding the cues of preterm infants is often more challenging than interpreting cues of full‐term infants. Preterm infants typically display decreased alertness and struggle with behavior regulation, which results in less clear cues for caregivers to interpret (Case‐Smith et al., 1998; Goldberg & DiVitto, 1995). These mixed behavioral cues can be particularly difficult for mothers to understand, leading them to seek more information about their infants, especially concerning hospital equipment, health, sensitivity to cues, and caregiving strategies (Eamkusolkit et al., 2017; Hesham et al., 2016; Sarapat et al., 2017). A previous qualitative study in Thailand found that mothers of preterm infants in the NICU often lacked sufficient knowledge about caring for and interacting with their infants, particularly during the initial days of separation (1–10 days). This knowledge gap is especially pronounced among young mothers with low educational levels or working mothers (Eamkusolkit et al., 2017). Furthermore, information on how to care for preterm infants can vary according to the perspectives of the individual nurses and is often delayed; given when infants become stable. Understanding their maternal role is associated with improved maternal emotional closeness to the infant (Lebel et al., 2022). Consequently, the absence of timely information can increase mothers’ anxiety and fear in handling their preterm infants (Eamkusolkit et al., 2017; Fernández Medina et al., 2018; Sarapat et al., 2017; Sikorova & Kucova, 2012). This heightened anxiety negatively affects their psychological well‐being and impairs the mother–infant bonding process (Wigert et al., 2014). Therefore, early dissemination of information from the nurse regarding preterm infants’ cues and maternal roles during the infants’ hospital admission is essential to help mothers develop a bond with their infants.

### 1.2 The impact of prematurity and separation on the mother–child relationships

The emotional bond between mothers and their infants is a crucial aspect of the mother–infant relationship that markedly impacts attachment (Ettenberger et al., 2021). Bonding refers to the maternal perception of affection tie that mothers feel toward their infants, demonstrated through their desire to care for and not abandon their infants (Klaus & Kennell, 1976). Prolonged separation from their infants during the early postnatal period can adversely affect the mother–infant relationship, leading to a lack of emotional closeness (Flacking et al., 2012; Lomotey et al., 2019; Thomson et al., 2020), diminished attachment behaviors (Feldman et al., 1999; Pennestri et al., 2015), and an impaired bonding process (Flacking et al., 2012). Previous research has indicated that maternal bonding declined linearly with prolonged separation of mothers from the infants (Feldman et al., 1999). The separation results in a lack of maternal interaction, which is essential for forming a bond. The frequencies of maternal involvement in infant care, holding, and skin‐to‐skin contact are associated with emotional closeness. According to Väliaho et al. (2023), mothers reported that caring for and being physically close to their premature infants were vital for establishing a strong bond. The mothers of preterm infants often experience challenging emotions, such as feelings of failure and guilt, which complicate their ability to cultivate a maternal role and bond with their babies (Al‐Maghaireh et al., 2016; Fernández Medina et al., 2018; Spinelli et al., 2015). Furthermore, when mothers are concerned about the survival of their premature infants, they may emotionally distance themselves, hindering them from connecting to their infants (Carton et al., 2020; Flacking et al., 2012; Spinelli et al., 2015). This emotional distance acts as a barrier to participating in the care of the infants, and the lack of physical contact increases the risk of developing a poor mother–infant bond. The disruptions in mother–infant bonding can lead to a long‐term impairment of the mother–infant relationship, including infant maltreatment and neglect, as well as an increased likelihood of developing psychopathological disorders in adulthood, which poses significant future concern (Enns et al., 2002; Nakano et al., 2019). Similarly, mothers who struggle to bond with their infants, particularly those with preterm infants, often exhibit low coherence, which is characterized by a difficulty in accepting the needs of their preterm infants, including unrealistic fear of losing them, resulting in overprotective parenting styles in the future (Fernández Medina et al., 2018; Ncube et al., 2016).

Previous studies have suggested interventions to support nurturing touch and proximity between mothers and preterm neonates, such as skin‐to‐skin contact and massage, to improve mother–infant bonding (Cho et al., 2016; Hayeese et al., 2014; Shoghi et al., 2017). However, infants in the NICU often have complex medical conditions, including sensitivity to stimulation (Patteson & Barnard, 1990). Thus, nurses must assist mothers in understanding their preterm infants’ cues and promoting positive interactions by explaining how to avoid harmful stimuli and establish closeness, particularly during the initial week of their visit (Phuma‐Ngaiyaye & Welcome Kalembo, 2016; Sarapat et al., 2017). Several studies have underscored the importance of nursing support in enabling mothers to overcome their fear and develop an emotional connection with their infants. Benzies et al.’s (2013) systematic review found that maternal education and psychosocial support led to improved outcomes for mothers of preterm infants. Heo & Oh (2019) and Phianching et al. (2020) have explored the impact of educating mothers about the characteristics of preterm infants, their cues and behaviors, as well as the appropriate responses to these cues. Understanding their infants’ cues and behaviors helps mothers feel more connected to their infants, enhances their caregiving skills, and strengthens the bond between them (Huenink & Porterfield, 2017). Additionally, psychological support from nurses focuses on helping mothers explore and reflect on their feelings and perceptions regarding bonding, providing emotional support, and encouraging quality interaction between mothers and preterm infants to facilitate bonding. Eamkusolkit (2016) has previously implemented a coaching model to help mothers explore and reflect on their feelings about bonding. This coaching model aimed to foster an appropriate mindset that facilitates a healthy and positive bonding behavior, enabling mothers to respond effectively to their preterm infants’ cues. Similarly, Seiiedi‐Biarag et al. (2021) utilized a counseling method to explore mothers’ expectations of their infants and establish mutual goals concerning bonding, including clarifying the circumstances surrounding preterm birth to facilitate understanding. This emotional support helps mothers cultivate emotional stability as they bond with their preterm infants. When mothers experience reduced stress and gain insight into their emotions, they are better prepared to engage with their infants, thereby strengthening the mother–infant bond. Material support is crucial for maintaining the bond between mothers and their preterm infants, especially when physical distance is an issue. Recent studies have explored the use of technology, such as webcams, recorded parent voices, and telenursing, to address this challenge. However, the effects of these technologies are varied and data on mother–infant bonding remain limited. Telehealth initiatives have been implemented to provide mothers with information about their preterm infants and the hospital environment, aiming to improve attachment and reduce the stress associated with having preterm infants (Jafarzadeh et al., 2019). This approach allows mothers to access information at their own pace, rather than feeling overwhelmed by one‐time verbal communication. The virtual visitation model, which involves the use of webcams, enables mothers to have continuous access to their hospitalized infants, to interact with them in real time, and to be present at their bedside. This technology allows mothers to soothe their babies during painful procedures and to stay informed about their infants’ treatment. It helps mothers maintain a connection with their infants and continue their bond, even after being discharged from the postpartum unit; this is an especially important factor when physical visitation is not feasible (Dunham and Marin, 2020; Gibson & Kilcullen, 2020; Kerr et al., 2017).

In Thailand, even after the pandemic has ended, mothers whose infants are in the NICU often receive inconsistent support when bonding with their infants. Many mothers struggle to establish this bond because they lack opportunities to be with their infants in the NICU, as they need to return to work or care for their other children. Additionally, geographical barriers often prevent mothers from visiting their infants, as many mothers live far from the hospital. Therefore, there is a pressing need to develop interventions that can be support these mothers remotely, emphasize in helping mothers understand their premature infants’ cues, maternal roles and strengthen their bond. Thus, the present study aimed to assess whether an online intervention can improve mothers’ knowledge of preterm infants’ cues, and their role during their infants’ hospitalization, as well as can enhance mother–infant bonding.

## 2 METHODS

### 2.1 Procedures

Mothers in the hospital unit were recruited to participate in the study after the babies were born and admitted to the NICU or intermediate care unit (IMCU). The eligibility criteria included being at least 18 years old, having a single preterm infant, being fluent in Thai, capable of using a smartphone with internet access, and having made at least one visit to their infants in the NICU/IMCU. Mothers with a history of substance abuse, postpartum blues, psychiatric disorder, or visual or hearing impairment, those who stayed overnight with their infants in the NICU/IMCU, or those whose infants received sedative drugs were excluded from the study. Participants were informed about the research project and invited to participate for 7 days at no financial cost. The mothers were informed about the study objectives, potential benefits and risks, meeting schedules, activities during the intervention, and the protection of their rights as human study participants. This included the right to withdraw from the study at any time without facing any repercussions. Additionally, the mothers consented to have video calls with their infants, with the understanding that these sessions would not be recorded and that only the participants and researchers would have access to the calls according to the personal data protection (PDPA) regulation in Thailand. The first 38 mothers who provided consent were assigned to the control group, where they received standard care. Once all the infants in the control groups were discharged from NICU/IMCU, the research team approached the mothers whose infants had the same gestational age as those in the control group. Mothers who consented were then assigned to the experimental group. The researchers adhered to all regulations and guidelines established by the Institutional Review Board, Faculty of Nursing, Mahidol University, considering as the memorandum of understanding (MOU) with Siriraj Institutional Review Board (SIRB), Faculty of Medicine, Siriraj Hospital, with the certificate of approval (MU‐MOU CoA) numbered IRB‐NS2022/706.0308 and registration with the Thai Clinical Trials Registry (TCTR20230805001).

**FIGURE 1:**
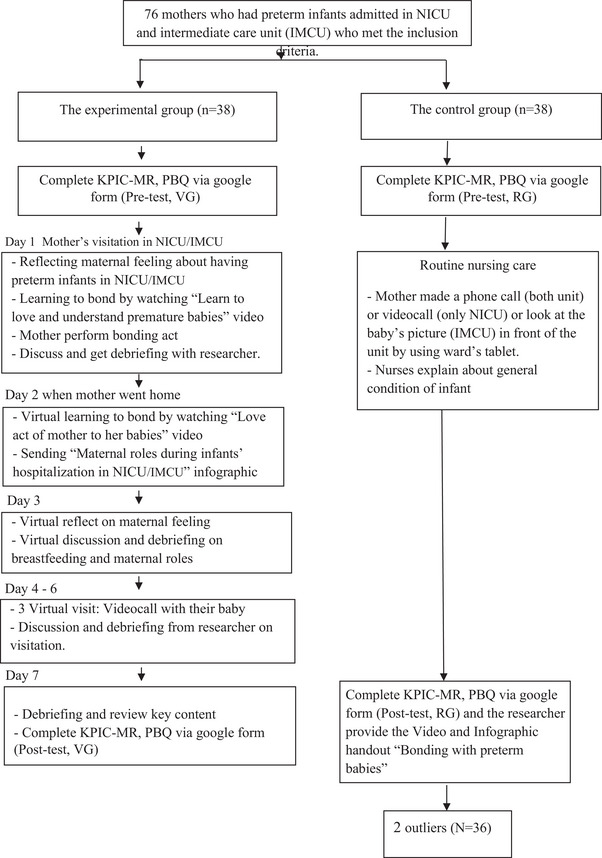
Data collection process.

After obtaining written consent from the mothers to participate in the study, the research commenced when the mothers visited their infants for the last time before being discharged from the postpartum unit and returning home, while their babies remained in the hospital. The mothers were asked to complete the pre‐ and posttest self‐report surveys. Figure 1 outlines the study procedures.

### 2.2 Bonding with preterm infants intervention

The researcher implemented a virtual supportive intervention for mothers whose infants remained in the hospital while they returned home. The intervention comprised seven sessions. The first session is in‐person, followed by six virtual sessions. The virtual sessions were conducted through a mobile application that included a messaging channel, both text and voice, as well as phone and video calls, video kits, and infographics. Through this application, mothers received daily notifications about their preterm infants’ condition and behaviors, along with access to videos and reading materials. The media developed by the researchers included two videos and infographics focusing on preterm infants’ cues and behaviors, and another covering breast milk pumping and mothers’ roles (bathing and changing diapers). The researcher obtained permission to record a preterm infant to teach mothers about recognizing preterm cues and behaviors, as well as the proper bathing and diaper‐changing techniques. The mother of that infant was informed that the video will only be used for ’research purposes only. A breastmilk pumping video was recorded by using a manikin and hospital equipment, with permission from the hospital to record and make media for ‘research purposes’ only. All the participants in the experimental group watched the same video and practiced reading infant cues when video calling with their infants.

The first session was conducted after the mothers were discharged from the postpartum unit. The researcher scheduled a one‐on‐one session with the mothers in the neonatal unit before they returned home while their infants remained in the hospital. During this time, the mothers were asked to reflect on their feelings regarding having premature infants for 5–10 min. Afterward, they watched a video demonstrating the characteristics of preterm infants, stability and stress cues, and appropriate responses to those cues. After watching the video, the researcher assisted the mothers in visiting their infants, encouraging them to notice the infants’ cues and to respond accordingly. The reflective session took place after the visit. Sessions two through seven were conducted using an application called “Bonding with preterm infants’ official Line account.” The mothers were prompted to share their feelings after returning home, receive daily information about their babies’ condition, and watch videos on topics such as breast milk expression, tub bathing, and diaper changing. Additionally, reflective opportunities regarding their feelings and clarification of any concerns were provided through the message box in the application. Video calls were scheduled at the mothers’ convenience, occurring at least three times within a 7‐day intervention period. These calls were initiated during regular visits, when babies received milk, and during tub baths. During the video calls, mothers were asked to interpret their babies’ cues and received suggestions on how to positively interact with their infants. A mini camera was positioned outside the incubator or crib to show real‐time videos of the babies according to a schedule arranged between the researchers and participants.

Contrarily, the control group receive a standard routine ward care. The control group was discharged from the postpartum unit and returned home, similar to the experimental group. They were informed about their preterm infants’ condition only if they contacted the ward. The mothers of preterm infants in the IMCU either received photographs of their infants or were informed of their condition. The mothers in the NICU could request video calls, but the duration of these calls depended on the nurse's availability. Each mother could see her baby using a hospital's device once a day for approximately 5–10 min, with no further instructions or explanations regarding the infant's general condition provided by the nurses in the unit.

At the end of the intervention, both groups were asked to complete the Knowledge Related to Preterm Infants’ Cues and Maternal Roles (KPIC‐MR) questionnaire and Postpartum Bonding Questionnaire (PBQ) as a posttest. The control group then subsequently had access to the same videos and infographics that the intervention group had during their sessions.

### 2.3 Participants

The participating mothers (*N* = 74) were in their early 30s (age: M = 32.03 years, standard deviation [SD] = 5.71), had at least a middle school education, lived with their husbands in a nuclear family, and had another child at home, in addition to the infant they intended to raise. The mothers had sufficient family income to meet their daily needs and lacked any prior experience with preterm care. The detailed in Tables 1 and 2, which present the characteristics of the full sample and each mother group.

**TABLE 1:** Maternal demographic characteristics.

Characteristics	Full group (*N* = 74)		Experiment group (*N* = 38)		Control group (*N* = 36)		χ^2^
	*N*	%	*N*	%	*N*	%	
**Educational level**							1.19
Primary school or lower	6	8.1	3	7.9	3	8.3	
Middle school	25	33.8	12	31.6	13	36.1	
Vocational education	6	8.1	4	10.5	2	5.6	
Bachelor's degree	29	39.2	14	36.8	15	41.7	
Master's degree or above	8	10.8	5	13.2	3	8.3	
**Marital status**							4.01
Couple and live together	68	91.9	33	86.8	35	97.2	
Separate	4	5.4	4	10.5	0	0	
Divorce	2	2.7	1	2.7	1	2.8	
**Family income**							.86
Not enough and having debt	9	12.2	5	13.2	4	11.1	
Not enough but does not have any debt	2	2.7	1	2.6	1	2.8	
Enough but does not have any saving	33	44.6	15	39.5	18	50	
Enough and can do the saving	30	40.5	17	44.7	13	36.1	
**Types of family**							1.00^a^
Nuclear	47	63.5	24	63.2	23	63.9	
Extended	27	36.5	14	36.8	13	36.1	
**The feeling toward current infant**							1.00
Pleasant	74	100	38	100	36	100	
**Experience with preterm care**							1.00^a^
Yes	19	25.7	10	26.3	9	25	
No	55	74.3	28	73.7	27	75	
**The intention of raising the infant**							.67
Raise by yourself	53	71.6	28	73.7	25	69.4	
Get help from family member	18	24.3	8	21.1	10	27.8	
Hire caregiver	3	4.1	2	5.2	1	2.8	

^a^ Fisher's exact test

**TABLE 2:** Maternal demographic characteristics.

Characteristics	Full group (*N* = 74)		Experimental group *(N* *=* 38)			Control group *(N* *=* 36)			Z
	M	SD	Range	Mean	SD	Range	Mean	SD	
Age (years)	32.03	5.71	19–40	31.26	5.52	21–43	32.83	5.88	.24^t^
Number of family member	4.36	1.65	2–8	4.45	1.54	2–8	4.28	1.78	***−***.62
Number of children under care	.77	.80	0–3	.97	.82	0–3	.56	.74	**−**2.36^*^
The frequency of visitation									
Face‐to‐face	1.72	1.78	1–6	1.58	1.27	1–14	1.86	2.20	**−**1.26
Virtual	.85	1.64	0–4	.39	.89	0–8	1.33	2.07	**−**2.35^*^

*Note*: t = *t*‐test, ^*^ *p* < .05.

The participating infants were, on average, born at approximately 32 weeks (gestational ages: M = 32.11 weeks, SD = 2.76) and weighed an average of 1904 grams at birth (weight at birth: M = 1904.5 years, SD = 461.49). The majority of these infants were males and were delivered via cesarean section. They experienced respiratory distress and received breastmilk. The various health conditions of the infants are detailed in Tables 3 and 4, which present the characteristics of the full sample and each infant group.

**TABLE 3:** Infant's characteristics.

Characteristics of infants	Full group (*N* = 74)		Experiment group (*N* = 38)		Control group (*N* = 36)		χ^2^
	*N*	%	*N*	%	*N*	%	
**Sex**							.34^a^
Female	28	37.8	12	31.6	16	44.4	
Male	46	62.2	26	68.4	20	55.6	
**Types of delivery**							.38^a^
Normal labor	14	18.9	9	23.7	5	13.9	
Cesarean	60	81.1	29	76.3	31	86.1	
**Current diagnosis**							6.84
Respiratory distress	42	56.8	21	55.3	21	58.3	
TTNB	19	25.7	12	31.6	7	19.4	
BBA	2	2.7	1	2.6	1	2.8	
Cardiovascular system	2	2.7	2	5.3	0	0	
**Types of feeding**							1.37
Breastmilk	38	51.4	22	57.9	16	44.4	
Formula milk	34	45.9	15	39.5	19	52.8	
Both	2	2.7	1	2.6	1	2.8	
**Types of oxygen**							16.99^*^
ETT	14	18.9	9	23.7	5	13.9	
CPAP	22	29.7	14	36.8	8	22.2	
Oxygen canular	7	9.5	7	18.4	0	0	
None	31	41.9	8	21.1	23	63.9	
**Medical equipment**							
IV catheter	21	28.4	12	31.6	9	25	.61^a^
UAC/UAV	18	24.3	9	23.7	9	25	1.00^a^
PICC line	6	8.1	3	7.9	3	8.3	1.00^a^
EKG monitoring	31	41.9	24	63.2	7	19.4	.00^a^,^*^
Phototherapy	7	95	2	5.3	5	13.9	.26^a^
Incubator	65	87.8	35	92.1	30	83.3	.30^a^
Crib	9	12.2	3	7.9	6	16.7	.30^a^
OG tube	71	95.9	36	94.7	35	97.2	1.00^a^
Pulse oximeter	64	86.5	37	97.4	27	75	.01^a^ ^*^

^a^ Fisher's exact test, ^*^ *p* < .05.

**TABLE 4:** Infant's characteristics.

Characteristics	Full group (*N* = 74)		Experiment group (*N* = 38)			Control group (*N* = 36)			z
	Mean	SD	Range	Mean	SD	Range	Mean	SD	
**Gestational ages at birth (weeks)**	32.11	2.76	28–36.6	31.97	2.91	28–36.3	32.26	2.62	−.43
**Weight at birth**	1904.05	461.49	1500–3850	1895.53	466.54	1310–3000	1913.06	462.53	−.08
**Current weight**	1915.07	456.39	1500–3840	1904.61	464.91	1310–3170	1926.11	472.22	−.07
**Day of life**	3.54	4.15	1–11	2.21	2.17	1–17	4.94	5.19	**−**1.93

### 2.4 Measures

The KPIC‐MR questionnaire was utilized to assess mothers’ understanding of the premature infant's cues and their roles during their infants’ hospitalization. This questionnaire was developed by researchers based on literature review of Als (1982) and Browne and Talmi (2005). The accuracy of the questionnaire was reviewed by five experts, including one neonatal pediatrician, three neonatal nurses, and one nursing instructor. The content validity index was .98, and the reliability score of the KPIC‐MR was calculated using the test–retest method with a 7 day interval, yielding a reliability score of .82. The KPIC‐MR questionnaire consisted of the following two sections: questions 1–15 focus on characteristics, cues, and behavior of preterm infants, and questions 16–25 focus on the mothers’ roles. Each item is rated using the following three response options: yes (1), no (0), and do not know/unsure (0). The total scores can range from 0 to 25, with higher scores indicating a better understanding of the preterm infant's cues and behaviors, as well as the mothers’ roles. The sample items related to the preterm infants’ characteristics were as follows: “The baby twists his trunk or covers his face with his hands, indicating that he is relaxing”; “When a baby jerks his arms and legs or has splayed fingers, it means he is experiencing stress”; and “When the baby is fussy and has a grimace expression, it means he needs comfort.” Furthermore, the items for as assessing a mother's knowledge of her role in the hospital were as follows: “To obtain a large amount of breastmilk, the mother squeezes from the areola to the nipple using her index and thumb”; “The mother can touch the baby's trunk or reposition the baby during the visit”; and “The mother can interact with the baby when he opens his eyes and extends his arms and legs.”

The PBQ (Brockington et al., 2001) was utilized to identify early problems in the mother–child relationship. It consisted of the following four scales: general impaired bonding, rejection and pathological anger, infant‐focused anxiety, and incipient abuse. The PBQ comprised of 25 items, each scored on a 6‐point Likert scale ranging from 0 to 5. The total scores range from 0 to 125, with low scores indicating strong mother–infant bonding and high scores reflecting problematic bonding. A score of ≥26 suggests a bonding disorder, whereas scores of ≥40 indicate severe bonding disturbances. The questionnaire was originally developed by Brockington et al. (2001) and translated into Thai by Phianching et al. (2020). The original English version of PBQ has a Cronbach's alpha reliability of .87 and .95, .95, .93, .77 for the four scales. Its validity was .93. The reliability of the original Thai version of PBQ was .73. The researchers received permission from both original developers to use this questionnaire and its Thai translation. In the present study, both of the validity and Cronbach's alpha reliability of PBQ was .80.

### 2.5 Data analysis

Demographic data were analyzed using descriptive statistics, and the demographic differences between the experimental and control groups were assessed using chi‐square tests, Fisher exact test, Mann–Whitney *U*‐test, and *t*‐test. Continuous demographic variables including the number of family members, number of children, frequency of visitation before the intervention, gestational age, day of life, and birth weight and current weight of the infants, were analyzed using the Mann–Whitney *U*‐test due to their abnormal distribution. During the data analysis, two outliers were identified; these infants had IMCU stay durations of 1–3 months, whereas the others remained for only 1–14 days. Consequently, the researcher removed these two samples from the control group as the longer IMCU stays could affect the frequency of mothers' visits prior to the study, which in turn impacts mother‐infant bonding. The final sample comprised a total of 74 infants, with 36 in the control group and 38 in the experimental group.

The KPIC‐MR responses were analyzed using the Mann–Whitney *U*‐test for between group comparisons and Wilcoxon signed‐rank test for within‐group comparisons. Non‐parametric statistics were used due to the abnormal data distribution. These tests were performed to evaluate the statistical differences in the mean scores between the pretest and posttest data. An Independent *t*‐test was conducted to examine group differences in mean score for mother–infant bonding, while. Paired *t*‐test was used to analyze the difference in mean scores between the pretest and the posttest.

## 3 RESULTS

### 3.1 Preliminary analyses

The two groups had similar sociodemographic characteristics; however, the notable demographic difference was that the experimental group comprised mothers with more children in their families (z = **−**2.36, *p* < .05), whereas the control group reported more virtual visits (z = −2.35, *p* < .05). The infants in the experimental group also had more complex health conditions, as indicated by the type of oxygen used (χ^2^ = 16.99, *p* < .05) and the kinds of medical equipment required. Specifically, there were significant differences in EKG monitoring data (χ^2^ = .00, *p* < .05) and usage rate of a pulse oximeter (χ^2^ = .01, *p* < .05). Tables 1 and 2 present the demographic characteristics of the mothers in the full sample and in each group, whereas Tables 3 and 4 display the demographic characteristics of the infants.

The two groups showed similar results in terms of their pretest knowledge of the cues related to preterm infants and the mother's roles (KPIC‐MR; z = .09, *p* > . 05), They also exhibited similar scores in terms of the preterm infants’ cues and behaviors (z = 1.26, *p* > .05). However, there was a significant difference in terms of the knowledge of the mothers’ roles (z = 3.52, *p* < .05). Regarding the bonding between the mother and preterm infants, the groups were comparable in the scores in all aspects (total *t* = 1.03, *p* > .05) as well as in the general impaired bonding (*t* = 1.00, *p* > .05), rejection and pathological anger (*t* = .43, *p* > .05), infant focused anxiety (*t* = .03, *p* > .05), and incipient abuse (*t* = 1.39, *p* > .05) items. Tables 5 and 6 present the pretest scores on the knowledge of infant cues and mother–infant bonding for the full sample and each group, respectively.

**TABLE 5:** Comparison of mean knowledge scores in preterm infant cues and mothers’ roles.

Dimension	Full group (*N* = 74)		Experiment group (*N* = 38)		Control group (*N* = 36)		z
	M	SD	M	SD	M	SD	
**Total knowledge **							
Pretest	15.51	3.72	15	3.97	16.06	3.41	.09
Posttest	19.08	3.14	20.55	2.36	17.53	3.15	4.22^*^
Wilcoxon signed‐rank test (z)			5.32^*^		1.77		
**Aspect knowledge**							
1. Preterm cues and behavior							
Pretest	9	2.55	8.55	2.84	9.47	2.16	1.26
Posttest	11.53	2.26	12.42	1.83	10.58	2.31	3.78^*^
Wilcoxon signed‐rank test (z)			4.88^*^		1.76		
2. Mothers’ roles							
Pretest	7.55	1.44	8.13	1.21	6.94	1.41	3.52^*^
Posttest	7.55	1.44	8.13	1.21	6.94	1.41	3.52^*^
Wilcoxon signed‐rank test (z)			.00		.00		

^*^ *p* < .05.

**TABLE 6:** Comparison of mean scores in mother–infant bonding.

Dimension	Full group (*N* = 74)		Experiment group (*N* = 38)		Control group (*N* = 36)		*t*
	M	SD	M	SD	M	SD	
**Total mother–infant bonding **							
Pretest	16.66	9.31	15.58	6.15	17.81	11.74	1.03
Posttest	12.41	8.23	10.42	6.09	14.50	9.69	**−**2.18^*^
**Paired *t*‐test**			**−**5.08^*^		−1.21		
**Aspect mother–infant bonding**							
1. General impaired bonding							
Pretest	4.01	5.01	3.45	3.20	4.61	6.38	1.00
Posttest	4.01	5.01	3.45	3.20	4.61	6.38	**−**1.00
**Paired *t*‐test**			—		—		
2. Rejection and pathological anger							
Pretest	5.78	3.11	5.63	2.45	5.94	3.70	.43
Posttest	4.27	2.76	3.50	2.31	5.08	2.98	**−**2.56^*^
**Paired *t*‐test**			**−**4.68^*^		1.07		
3. Infant‐focused anxiety							
Pretest	5.82	2.55	5.82	2.11	5.83	2.97	.03
Posttest	4.93	2.34	4.66	2.08	5.22	2.59	1.04
**Paired *t*‐test**			**−**3.36^*^		.93		
4. Incipient abuse							
Pretest	.97	1.84	.68	1.60	1.28	2.05	1.39
Posttest	.38	1.19	.29	1.25	.47	1.13	.66
**Paired *t*‐test**			1.43		2.03		

^*^ *p* < .05, cannot be computed because the difference in mean scores was 0.

### 3.2 Comparison of the mean scores for the knowledge of preterm infant cues and mothers’ roles

The results of the Wilcoxon‐signed rank tests indicated that the experimental group showed significant improvements in total knowledge, as well as in knowledge of cues and behaviors, whereas the control group showed no significant improvements. There was no change in knowledge of mothers’ roles between pretest and posttest scores for either group. Additionally, Mann–Whitney *U*‐tests revealed that, at the posttest, the experimental group scored significantly higher than the control group in total knowledge and knowledge of preterm infant cues and behaviors. Furthermore, there was significant improvement in knowledge of mothers’ roles between the experimental and control groups (see Table 5).

### 3.3 Comparisons of the mean scores in mother–infant bonding

Independent *t*‐test revealed that the experimental group displayed a significant difference in the scores for total mother–infant bonding and rejection and pathological anger when compared to the control group. The low scores on PBQ indicate a strong mother–infant bonding. Therefore, the mothers in the experimental group exhibited stronger mother–infant bonding than those in the control groups. A paired *t*‐test indicated that at the posttests, the experimental group showed significantly different scores in the total mother–infant bonding, rejection and pathological anger, and infant‐focused anxiety items. However, no significant differences were found in the scores for general impaired bonding and incipient abuse measures (see Table 6).

## 4 DISCUSSION

The results of the present study indicate that our virtual support program is effective in enhancing mothers’ knowledge and bonding with their preterm infants. It provided consistent information to mothers regarding important infant cues and their maternal roles, along with psychological support, which improved mother–infant bonding. Individualized knowledge delivery, tailored to the mothers’ interests through the use of illustrations, written information, and opportunities for repetitive practice in reading infant cues and observing their infants’ behavior, remarkedly enhanced their understanding of the preterm cues and behaviors. Providing information that aligns with a person's interest makes the mother more eager to learn and more attentive. The preliminary analysis shows that all mothers in the study had positive feelings towards their current infants, showing interest and motivation to learn more about how to care for their infants. The method of delivering information is crucial; the study found that the use of illustrations and written materials was more effective than oral communication alone. Furthermore, the provision of information via videos and infographics correlated with a significant improvement in knowledge. This finding aligns with the results of Hesham et al.’s (2016) study, which demonstrated the effectiveness of using illustrated materials and written information for teaching mothers about preterm ’infants’ cues and behavior, as compared to oral instruction. Similarly, Maguire et al. (2007) have shown that maternal knowledge improved when mothers were taught about preterm cues using photobooks. The mothers of preterm infants often face overwhelming amounts of verbal information while navigating the challenges of their ’infants’ hospitalization. Therefore, a platform that allows mothers to process and learn information at their own pace is vital, as it aids in better knowledge retention. The opportunity for mothers to practice reading cues and to repeat this skill throughout the program is also essential, as repeated exposure to information enhances knowledge retention. This was confirmed in a study conducted by Browne & Talmi (2005), which revealed that mothers in the experimental group with preterm infants born before 32 weeks who received repeated information and had opportunities to practice reading cues exhibited a significantly higher understanding of preterm infant cues as compared to those in the control group. However, in the present study, the participants’ ability to fulfill their maternal roles was hindered by the COVID‐19 pandemic restrictions, which limited their opportunities to carry out these responsibilities. Proficiency in psychomotor skills develops with practice, making it essential to further enhance mothers’ knowledge of their roles in caring for preterm infants. The present study also revealed that the mothers in the experimental group, who had previous childcare experience exhibited significantly higher pretest knowledge scores than the first‐time mothers in the control group. This indicates that hands‐on experience in childcare, for instance, bathing and changing diapers, enhances mothers’ understanding of their roles.

The consistent early support provided to mothers significantly improved their bonding with their premature infants. This was noteworthy given that the infants in the experimental group faced more health complications, and the mothers had very limited prior virtual contact as compared to those in the control group. The results highlight the importance of early support in the following four aspects: informational, emotional, instrumental, and appraisal support. The mothers in the experimental group received information that helped them understand their infants’ behaviors. This newfound understanding shifted their perceptions of pregnancy into a recognition of the unique needs of preterm infants. They learned that these preterm infants exhibited unique behaviors requiring a different approach than what they expected during pregnancy. Additionally, the mothers learned to observe their preterm infants and respond appropriately to their cues, which facilitated a deeper understanding of their behaviors. With this information about the behaviors of preterm infants, mothers enhanced their responses, fostering a stronger sense of belongingness and bond with their infants This finding aligns with the results of Heo & Oh's (2019) and Bostanabad et al.’s (2017) studies, which reported marked enhancement in bonding when mothers of preterm infants received information about the behaviors of their infants and effective caregiving strategies. These include guidance on navigating the NICU environment, interpreting infants’ signals, and understanding the preterm infants’ sleep cycles. Our study results confirm the findings of a previous study, demonstrating that mothers who received similar interventions reported lower scores for rejection and pathological anger. This indicates that the mothers became more accepting of the premature infants’ condition and fostered a stronger sense of belongingness, enhancing the mothers’ bond with their infants after the intervention.

The emotional and appraisal support provided to the mothers offered them emotional stability, reducing their anxiety about the well‐being of their preterm infants and the uncertainties surrounding their situations. This support allowed mothers to express their concerns and receive clarification about the condition of their premature infants. The significant difference between the pre‐test and posttest scores for infant‐focused anxiety among the mothers who participated in the program indicated that straightforward explanations addressing their need for information about their infants significantly alleviated their anxiety related to unknown circumstances. This, in turn, resulted in positive maternal interactions with their infants, characterized by increased responsiveness, care, and affectionate holding. These findings align with previous studies that utilized coaching models and counseling methods, which emphasized the importance of exploring the feelings of mothers with preterm infants, reflecting on those feelings, and setting bonding goals. These approaches have shown significant results in enhancing mother–infant bonding (Eamkusolkit, 2016; Seiiedi‐Biarag et al., 2021). The study found no significant differences in the scores for infant‐focused anxiety between the experimental and control groups, which may be attributed to the fact that the infants in the control group experienced less complex health issues as compared to those in the experimental group. This indicates that the degrees of prematurity may affect the quality of attachment. Secure attachment is notably more prevalent among infants with higher gestational age, greater birth weight, and less complex medical conditions (Fuertes et al., 2024).

Having alternative options for mothers who cannot visit their infants in the hospital allowed them to maintain an emotional connection with their infants. The virtual support program provided online messaging, video calls, and voice notes as alternatives for mothers to interact with their infants while they were physically absent. Consistent assistance is offered for their preterm infants, along with emotional support and positive reinforcement to promote maternal engagement in observing and interpreting their ’infant's signals. This engagement leads to better mother–infant bonding. The effectiveness of this support mechanism on mother–infant bonding was also evident in telenursing and the use of webcams. Jafarzadeh et al. (2019) have noted the significant impact of using telephone communication to provide information about the NICU environment, the behaviors of preterm infants, their growth, breastfeeding abilities, and overall preterm care, as well as, counselling for mothers of preterm infants. Their study found a notable improvement in attachment as a result of using telephone communication. Similarly, Gibson & Kilcullen (2020) attached webcams to infants’ bedsides, enabling mothers to visit virtually from their homes. These tools help mothers stay connected with the infant, thereby strengthening their feelings of closeness and increasing parental responsiveness (Kerr et al., 2017; Dunham and Marin, 2020).

## 5 CONCLUSIONS

This study demonstrates that a virtual program can effectively provide support to mothers of premature infants to improve their understanding of their roles and their preterm infant's cues and bonding. Understanding these cues and receiving emotional support is crucial for fostering emotional closeness between mothers and their preterm infants. Initiating support from the first day of mothers visit their premature infants and ensuring its consistency are essential for strengthening the bond between mothers and infants. Virtual communication technology serves as an alternative for mothers who are unable to visit their preterm infants in the hospital. It includes features such as video calls, instant messaging, videos and infographics.

## 6 IMPLICATIONS FOR PRACTICE AND/OR FURTHER RESEARCH

Nurses and healthcare providers can utilize technology to support mothers who are unable to visit their infants in the hospital. This support should begin during the mother's first visit and encompass four key aspects: providing knowledge about the cues of preterm infants and their roles, informing mothers about their infants' conditions daily, and offering emotional, appraisal, and material support. The technology should be an easily accessible platform that helps ensure consistency in the support provided. The current study indicates that the COVID‐19 pandemic has impacted mothers' knowledge of their roles due to visitation restrictions. This finding suggests that, while virtual communication is valuable, hands‐on experience remains crucial. Therefore, future research should consider a hybrid intervention that combines both virtual support and in‐person interactions to enhance maternal knowledge and skills in caring for their babies. It is important to note that this study employed a quasi‐experimental design, utilizing matching rather than randomization to assign participants to either the intervention or control group. Additionally, there were some exogenous factors that could not be controlled, leading to a heterogeneous demographic among the participants. These factors included differences in infants' health conditions, the frequency of maternal virtual visits prior to the intervention, and the number of children under care before the intervention. Such variations may have influenced the outcomes. Therefore, future studies should consider controlling for these factors and using a randomized controlled trial to assess mother‐infant bonding 1 month after the program's completion to examine long‐term outcomes. Since mother‐infant bonding is an ongoing process, this approach would help better understand its evolution over time.

## CONFLICT OF INTEREST STATEMENT

The authors declare there is no conflict of interest.

## Data Availability

Data supporting the findings of this study are available from the corresponding author upon reasonable request.
